# Syncope as the initial symptom in a patient with nasopharyngeal carcinoma: a case report

**DOI:** 10.1186/s12872-023-03174-2

**Published:** 2023-03-14

**Authors:** Lin-lin Zhang, Lei Wang, Jie Ge, Shi-kun Sun

**Affiliations:** 1 Huantai County People’s Hospital, No. 2198 Huantai Avenue, Suo Town, Huantai County, Zibo City, Shandong Province China; 2 The First Affiliated Hospital of Soochow University, Suzhou, Jiangsu Province China

**Keywords:** Syncope, Nasopharyngeal carcinoma, Carotid sinus syndrome

## Abstract

**Background:**

A high prevalence of nasopharyngeal carcinoma (NPC) has been found in China, but it rarely occurs with syncope. Studies have demonstrated that syncope due to NPC may be related to carotid sinus syndrome, glossopharyngeal irritation, and parapharyngeal and retropharyngeal space lesions. Such patients require evaluation by nasopharyngoscopy and head magnetic resonance imaging/computed tomography. There is no known single effective treatment for these patients. Various interventions may be considered in an effort to relieve syncope, including vasoconstrictive drugs, cardiac pacemaker implantation, radiotherapy and chemotherapy, and surgical resection.

**Case presentation:**

This case report describes a 56-year-old man who developed recurrent syncope with atrial fibrillation, a long RR interval, and hypotension. A single chamber pacemaker was fitted, but it failed to relieve the symptom. Cranial magnetic resonance imaging and pathological tests led to a final diagnosis of NPC. After six courses of chemotherapy and 35 sessions of radiotherapy, the patient became asymptomatic. However, he died from a massive uncontrolled hemorrhage in the nasopharynx two years later.

**Conclusions:**

This case brings attention to the fact that syncope can be a symptom of NPC. Due to the insidiously malignant nature of this cancer, when a patient presents with syncope, clinicians should bear in mind this connection, albeit a rare one. There are at least two ways of treating NPC-associated syncope, but there is disagreement about which is the most effective.

## Background

Nasopharyngeal carcinoma (NPC) presents with a variety of signs and symptoms, such as painless cervical lesions, epistaxis, and conductive hearing loss. Syncope is normally unrelated to NPC. However, this report describes a 56-year-old man who developed recurrent syncope as a result of NPC and makes the point that rare causes of syncope should not be ignored because patients with NPC, which is an insidious malignancy, need to be identified in a timely manner.

## Case presentation

A 56-year-old man was admitted to Huantai County People’s Hospital, Shandong after experiencing syncope two to three times a day for two days. He had been suffering from a headache, dizziness, a sore throat, and hyperhidrosis and then fainted. The syncopal episodes lasted about 40 to 50 seconds each, and after he regained consciousness, he felt weak. The patient had a 3-year history of paroxysmal atrial fibrillation (AF) and had received intermittent oral amiodarone.

**Fig. 1:**
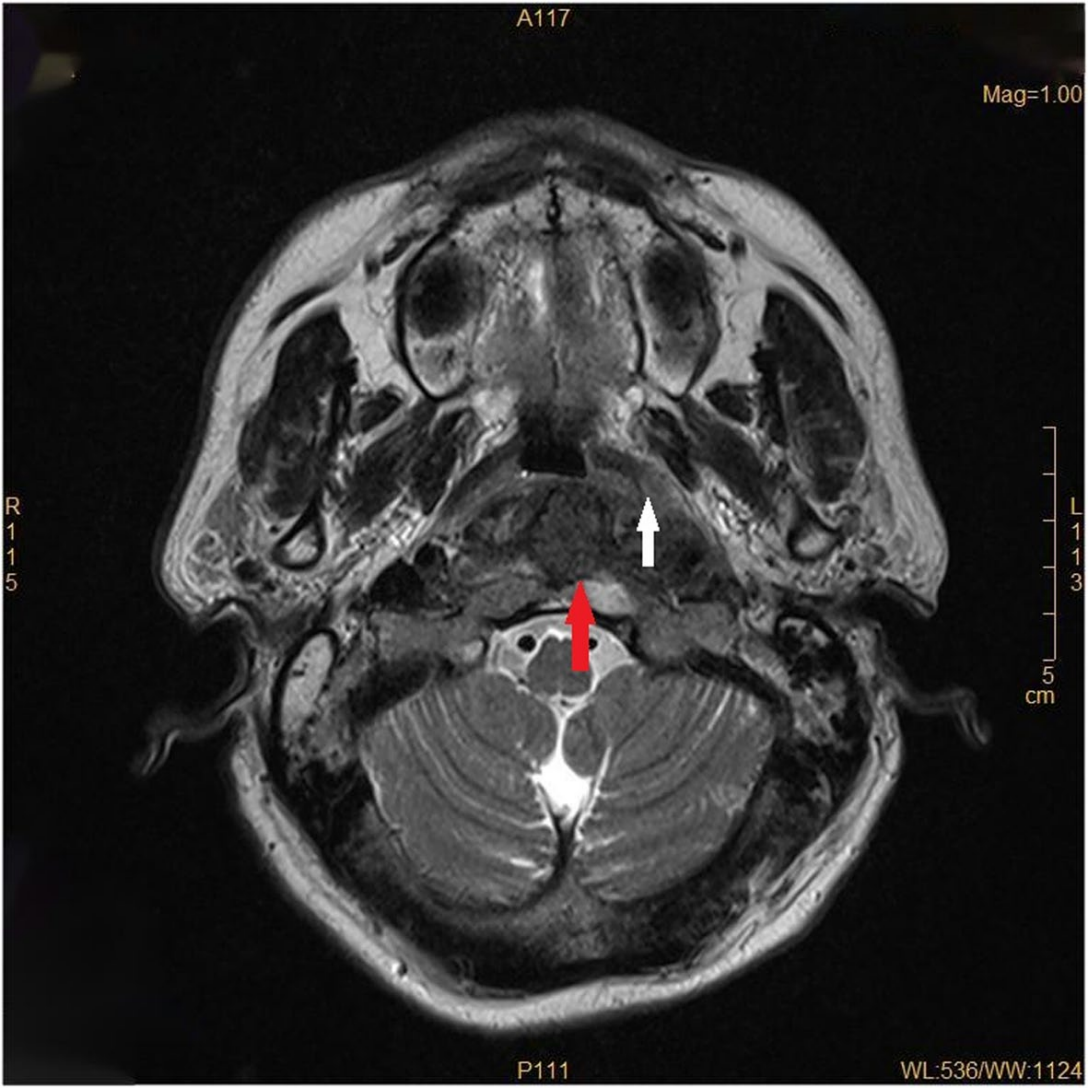
Thickening of nasopharyngeal soft tissue and posterior pharyngeal wall: Bone destruction is indicated by the red arrow, and narrowing of the local pharyngeal crypt and the eustachian tube is indicated by the white arrow

**Fig. 2:**
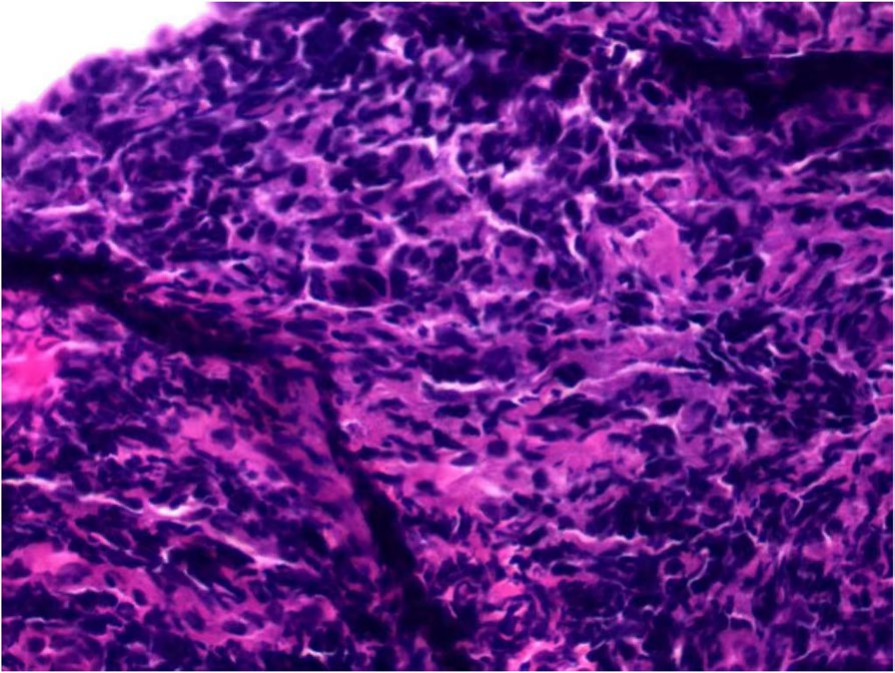
Squamous-cell carcinoma

On admission, the patient displayed AF with an average heart rate of 72 beats/minute and blood pressure (BP) of 87/61 mmHg. Physical and neurological tests showed no obvious abnormality. Electrocardiography (ECG) indicated AF rhythm, a long RR interval, and a junctional escape rhythm; transthoracic echocardiography was normal; and a 24-hour ECG Holter monitor detected AF rhythm with the longest RR interval of 1.8 seconds. A routine blood test and measurement of electrolytes, glucose, liver, kidney, thyroid function, and high-sensitivity troponin T were all negative. Cranial magnetic resonance imaging (MRI) revealed a thickened nasopharyngeal soft tissue and posterior pharyngeal wall, bone destruction, and narrowing of the local pharyngeal crypt and eustachian tube (Fig. 1). The diseased region (5.8 × 2.6 × 4.1 cm) was moderately enhanced after gadopentetic acid (Gd-DTPA) injection, with unclear demarcation from the surrounding structures, which conformed to the features of a tumor. Vascular imaging of cerebral arteries showed normal blood vessel branches and diameter. However, the consultation opinion of the provincial imaging research institute was that it was a pharyngeal abscess, so the patient was given amoxicillin and clavulanate potassium. On the ninth day of admission, the patient had another syncopal episode, and ECG monitoring showed AF rhythm with the longest RR interval of 3 seconds and BP of 88/60 mmHg, so a pacemaker was implanted. Five days after the operation, the patient experienced recurrent syncopal episodes associated with a pacing rhythm caused by a slow ventricular rate of AF and BP of 80/55mmHg, and he was transferred to Qilu Hospital in Shandong. Based on the results of cranial MRI and pathological tests (Fig. 2), NPC was diagnosed. The patient underwent six courses of chemotherapy (with cisplatin and fluorouracil) and 35 sessions of radiotherapy at Qilu Hospital, after which he remained asymptomatic except for a dry pharynx and sore throat. Two years later, he died from a massive uncontrolled hemorrhage in the nasopharynx. The timeline of his symptoms is shown in Table 1.

**Table 1:** The patient’s history

	In hospital	9 days later	14 days later	1-year follow-up	2-year follow-up
Symptom	syncope	syncope	syncope	remission	died
Rhythm	AF with 1.8 s	AF with 3 s	pacing rhythm		
Blood Pressure (mmHg)	87/61	88/60	80/55		

## Discussion and conclusions

Syncope refers to a transient loss of consciousness caused by cerebral hypoperfusion, and it is usually resolved spontaneously. There are three types of syncope: (1) nerve-mediated syncope, including vasovagal syncope, situational syncope, carotid sinus syncope, and atypical syncope; (2) orthostatic hypotensive (OH) syncope; and (3) cardiac-origin syncope. Although rare, syncope may occur in patients with NPC [1], and the underlying reasons for it may be parapharyngeal infiltration, invasion of the skull base, and involvement of the cranial nerve, according to a literature review on Google Scholar (Table 2).

**Table 2:** Similar published case reports

Numberof cases or sex/age	Symptoms	Risk factors for developing syncope	Treatment	Outcomes	Case link
**3 cases**	Syncope; neck masses, invasion of the skull base, and lower cranial nerve palsies	Mass compression of the carotid sinus or glossopharyngeal nerve invasion	Intravenous atropine followed by radiotherapy and/or chemotherapy		https://europepmc.org/article/med/8490777
**Male/62**	Syncope; hypotension, bradycardia, and long sinus pauses up to 2 s	Large parapharyngeal-space tumor enclosing the carotid sinus and arteries, jugular veins, and hypoglossal and vagus nerves	Pacemaker implantation; chemotherapy (with cisplatin and fluorouracil) combined with radiotherapy	No evidence of malignant disease found in nasal endoscopy and biopsy sample	https://www.thelancet.com/journals/lanonc/article/PIIS1470-2045(05)70172-8/fulltext
**2 cases**	Frequent syncopal attacks five months pre-diagnosis	Involvement of the glossopharyngeal or vagal nerve by para-pharyngeal extension of the tumor			https://www.sciencedirect.com/science/article/pii/0303846794900515
**Male/75**	Syncope; hypotension and reduced plasma norepinephrine (NE) levels	Carotid sinus hypersensitivity	Intravenous NE combined with oral midodrine	No further syncopal episodes; periodic occurrence of hypertension	https://www.ncbi.nlm.nih.gov/pmc/articles/PMC6156047/
**Male/53**	Syncope at the onset of sleep; cyclic variation of the heart rate; left facial dysesthesia, diplopia, dysphonia, and dysphagia	Recurrence of a nasopharyngeal carcinoma inducing fluctuation of vagal tone at the onset of sleep	Chemotherapy	No further syncopal attacks; cessation of cyclic variation of the heart rate	https://link.springer.com/article/10.1007/BF02767049
**Female/66**	Frequent and recurrent syncope for 2 weeks, 2 to 4 times per week; palpitation, dizziness, and neck tightness before the attacks, then the loss of consciousness; sinus bradycardia	Carotid sinus syndrome accompanied by arrhythmia	Chemoradiotherapy	Reduced left nasopharyngeal and carotid sheath space masses reduced in size; no recurrence of syncope for two years	https://www.frontiersin.org/articles/10.3389/fcvm.2021.796653/full
**Male/72**	Frequent and recurrent syncopal episodes lasting about 30 s each; palpitation, nausea, and dizziness, lasting for 3–4 s	Carotid-sinus hypersensitivity secondary to mechanical compression of the carotid sinus	Chemoradiotherapy	Significant reduction of neck masses; no recurrence of syncope for two years	https://www.internationaljournalofcardiology.com/article/S0167-5273(15)00439-8/fulltext
**Male/56**	Horner syndrome and syncopal episodes	Stimulation of the carotid sinus by the direct mass effect of the metastatic left submandibular lymph nodes and anterior cervical lymph nodes	Chemotherapy and radiation therapy	No syncopal attacks after cervical lymph node excision	https://journals.lww.com/theneurologist/Abstract/2012/07000/Nasopharyngeal_Carcinoma_Presenting_With_Horner.9.aspx
**Man/68**	Light-headed and nearly fainting; blurred vision and right arm tremors; hearing loss and otalgia in the left ear; low blood pressure; junctional rhythm	Carotid sinus syndrome	Intravenous and oral atropine sulfate; radiotherapy, oxygen therapy, and weekly infusions of perfluorocarbon emulsion	No recurrence of syncope or hearing deficit; reduced tumor size; complete resolution of carotid sinus syndrome; patient dead three months later	https://jamanetwork.com/journals/jamainternalmedicine/article-abstract/609899
**Man/69**	Frequent and recurrent syncopal episodes lasting 3–4 min each during stress or emotional upset	Right vagus nerve compressed by lymphadenopathy	Chemoradiotherapy	All syncope symptoms resolved	https://onlinelibrary.wiley.com/doi/abs/10.1111/j.1368-5031.2004.0044.x
**Man/50**	Intermittent fainting when tilting neck; bradycardia; hypotension	Tumor in left nasopharynx extended to left carotid sheath space	Chemoradiotherapy	Disappearance of nasopharyngeal and neck masses; no recurrence of syncope	https://link.springer.com/article/10.1007/BF01054725

Carotid sinus syndrome (CSS) occurs secondary to mechanical compression of the carotid sinus and is traditionally classified into three subgroups: the cardioinhibitory (CI) subtype (defined as asystole for > 3 s without a fall in arterial pressure), the vasodepressor subtype (defined as an isolated decline in systolic BP of > 50 mmHg), and mixed subtypes. The pathophysiology of CSS remains relatively obscure. Several pathophysiologic mechanisms have been considered, including atherosclerotic noncompliance, sternocleidomastoid proprioceptive denervation, and generalized autonomic dysfunction [2]. In one study, CSS in an NPC patient was accompanied by norepinephrine secretion deficiency, which might give rise to vasodilation [3]. NPC itself may not sensitize the carotid sinus; however, various biological and chemical mediators can do so [4]. Head and neck tumors cramming into parapharyngeal space can also result in the development of syncope, which is known as parapharyngeal space syncope syndrome [5]. The parapharyngeal space is close to the vagal, glossopharyngeal, and sublingual nerves; thus, a tumor may cause syncope by stimulating the vagus nerve or the glossopharyngeal nerve or both nerves to cause vasovagal or reflex syncope. In the present report, the syncope experienced by the patient was related to a long RR interval and hypotension, and cranial MRI and pathological tests led to a diagnosis of NPC. It was speculated that the patient’s syncope was caused by hypersensitivity of the carotid sinus or tumor-induced irritation of the glossopharyngeal nerve, both of which are afferent to the medullary vasodepressor region, leading to increased vagal tone and decreased sympathetic tone.

NPC-induced syncope is uncommon in clinic, but an early diagnosis determines the therapeutic outcome for patients. This specific condition should be distinguished from cardiac-origin syncope and OH syncope. The causes of cardiac syncope include arrhythmia and organic heart disease, and the diagnosis depends on ECG, transthoracic echocardiography, myocardial MRI, and coronary angiography. A diagnosis of OH syncope is based on BP variation in the supine or sitting position; the patient exhibits a decrease in systolic BP of 20 mmHg or in diastolic BP of 10 mmHg or a reduction in systolic BP to < 90 mmHg. The measurement of BP and heart rate in the supine and vertical positions facilitates the diagnosis of OH syncope.

Nasopharyngeal carcinoma is usually diagnosed after a patient presents with a seemingly unrelated complaint. Symptom recognition, awareness of risk factors, and timely referral to an otorhinolaryngology service are essential for early diagnosis and intervention. Imaging usually includes computed tomography and MRI, with studies favoring MRI for both staging and follow-up.

No consensus has been reached on the appropriate therapy for NPC-associated syncope. Two main treatments may be utilized, depending on the type of CSS. Firstly, midodrine serves as the main drug for vasodepressor CSS. Glucocorticoids, such as dexamethasone, increase blood volume through kidney sodium reabsorption, which can affect the sensitivity of baroreceptors. Glucocorticoids also enhance the effect of norepinephrine on vasoconstriction and, thus, reduce sympathetic activation, making them suitable for this type of vascular suppression. Secondly, a pacemaker implant, preferably a dual-chamber one, constitutes a common clinical practice for treating CI-CSS [6]. Carotid sinus denervation, carotid endarterectomy, and cardiac ganglion plexus ablation have also been identified as modalities for treating CSS [2, 7, 8]. Although promising, these approaches still need further validation, especially with respect to long-term effects. Syncopal symptoms can be improved after radiotherapy and chemotherapy [9], the mechanism of which might be related to tumor revision rather than only carotid sinus adjustment [10]. Because of the limited recognition of a relationship between NPC and syncope, the physician in the present case ignored such a possibility. The patient was fitted with a pacemaker that mitigated the syncope initially, but it recurred five days after the operation. The authors speculate that the vasopressor type of syncope relates more to tumor-associated CSS than CI does. After radiotherapy and chemotherapy for the NPC, this patient experienced no further syncopal episodes.

Syncope rarely occurs as the initial presentation of NPC. Carotid sinus syndrome is considered the potential intrinsic cause of syncope in patients with NPC. Hence, cranial imaging tests and nasopharyngoscopy may be needed for patients suspected of having NPC-associated syncope. Permanent pacing is often performed for CI-CSS. Patients with NPC who have CSS develop bradycardia and hypotension, so chemoradiotherapy may be a better choice for controlling their syncope. Physicians should attempt to identify the origin of recurrent syncope through an assessment of the patient’s clinical condition and the performance of relevant tests, which will facilitate early diagnosis, precise treatment, and an improved prognosis.

## Data Availability

All available information is contained within the present manuscript.

## Abbreviations

AF: Atrial fibrillation
BP: Blood pressure
CI: Cardio inhibitory
CSS: Carotid sinus syndrome
ECG: Electrocardiogram
MRI: Magnetic resonance imaging
NPC: Nasopharyngeal carcinoma
OH: Orthostatic hypotensive
